# A 3D CFD Simulation and Analysis of Flow-Induced Forces on Polymer Piezoelectric Sensors in a Chinese Liquors Identification E-Nose

**DOI:** 10.3390/s16101738

**Published:** 2016-10-20

**Authors:** Yu Gu, Yang-Fu Wang, Qiang Li, Zu-Wu Liu

**Affiliations:** 1 School of Automation and Electrical Engineering, University of Science and Technology Beijing, Beijing 100083, China; tslee@xs.ustb.edu.cn (Q.L.); lzw3113@126.com (Z.-W.L.); 2 School of Civil Engineering, Beijing Jiaotong University, Beijing 100044, China; 14120986@bjtu.edu.cn

**Keywords:** 3D-numerical simulation, polymer piezoelectric sensors, flow-induced forces, frequency fluctuation change

## Abstract

Chinese liquors can be classified according to their flavor types. Accurate identification of Chinese liquor flavors is not always possible through professional sommeliers’ subjective assessment. A novel polymer piezoelectric sensor electric nose (e-nose) can be applied to distinguish Chinese liquors because of its excellent ability in imitating human senses by using sensor arrays and pattern recognition systems. The sensor, based on the quartz crystal microbalance (QCM) principle is comprised of a quartz piezoelectric crystal plate sandwiched between two specific gas-sensitive polymer coatings. Chinese liquors are identified by obtaining the resonance frequency value changes of each sensor using the e-nose. However, the QCM principle failed to completely account for a particular phenomenon: we found that the resonance frequency values fluctuated in the stable state. For better understanding the phenomenon, a 3D Computational Fluid Dynamics (CFD) simulation using the finite volume method is employed to study the influence of the flow-induced forces to the resonance frequency fluctuation of each sensor in the sensor box. A dedicated procedure was developed for modeling the flow of volatile gas from Chinese liquors in a realistic scenario to give reasonably good results with fair accuracy. The flow-induced forces on the sensors are displayed from the perspective of their spatial-temporal and probability density distributions. To evaluate the influence of the fluctuation of the flow-induced forces on each sensor and ensure the serviceability of the e-nose, the standard deviation of resonance frequency value (SD_F_) and the standard deviation of resultant forces (SD_Fy_) in y-direction (F_y_) are compared. Results show that the fluctuations of F_y_ are bound up with the resonance frequency values fluctuations. To ensure that the sensor's resonance frequency values are steady and only fluctuate slightly, in order to improve the identification accuracy of Chinese liquors using the e-nose, the sensors in the sensor box should be in the proper place, i.e., where the fluctuations of the flow-induced forces is relatively small. This plays a significant reference role in determining the optimum design of the e-nose for accurately identifying Chinese liquors.

## 1. Introduction

Chinese liquors are well-known beverages all over the world. The consumed amount and value in 2014 were reported to be 75,282.7 million liters and $133.25 billion United States Dollars (USD) [1]. The enticing flavor profile for which Chinese liquors are so renowned is the result of the metabolic activity of a microbial community which provides thousands of micro-constituents such as esters, acids, phenols, and carbonyl compounds, etc. Chinese liquors are classified into 12 different flavors according to the content and type of the microconstituents [2]. The analysis of the aroma of Chinese liquors is an especially complex problem due to the components’ complexity and similarity. The traditional method of the analysis and identification of Chinese liquors is by professional sommeliers [3], but their accuracy and objectivity cannot be always ensured because sommeliers are affected by health conditions, emotions and the environment. Chromatographic [4] and spectroscopic [5] chemical analysis methods have been used to measure Chinese liquor products. However, these chemical analysis methods need to use large-scale devices and consume a lot of time, which could cause inevitable inconveniences for users. Taste sensors [6], systems composed of a sensor array and a data-processing unit, based on several transduction mechanisms such as voltammetry [7,8], conductivity [9] and potentiometry [10,11], can be used to identify liquid media containing multiple taste components. Detection equipment based on taste sensors could also be used to identify Chinese liquors, but the high cost of the equipment limits their broad application. The development of odor, vapor, and gas detection systems comprised of multi-sensor arrays together with pattern recognition techniques constituting so-called electronic nose systems is a fledgling area with applications in a variety of detection and identification problems [12]. Electronic nose systems have been used in the food industry for the inspection of food quality [13], control of cooking processes [14], and food flavor assessment [15], e.g., due to their objectivity, portability, accuracy, low cost and shorter analysis time. 

Our group has reported the design and application of a novel polymer piezoelectric sensor electronic nose (e-nose) [16]. The e-nose is used for quickly and easily summarizing Chinese liquor flavor characteristics and provides an objective method of communication between engineers and end-users. The e-nose is an instrument consisting of an array of reversible but only semi-selective gas sensors coupled with a series of pattern recognition algorithms. Gu et al. [17,18] used the e-nose to classify different kinds of Chinese liquors by developing two pattern recognition systems based on linear discriminant analysis (LDA), and a back propagation (BP) neutral network method. The identification accuracies of LDA, and BP in predicting outcomes were 93.3% and 98%, respectively. To improve the identification accuracy of the e-nose, these methods should be conducted not only by developing a pattern recognition system, but also capturing the raw data (the testing frequency values) accurately. Semi-selective gas sensors that incorporate two polymer detection layers and transform an interaction into a testing frequency signal are used as transducers [19]. The testing frequency values, the resonance frequency values of each sensor at stable state, are implemented to distinguish Chinese liquors [17]. Those frequency values decrease at first and then stabilize in the adsorption phase, corresponding to the quartz crystal microbalance (QCM) principle [16]. However, the principle fails to completely account for a particular phenomenon—we found that the resonance frequency values fluctuated in the stable state. The resonance frequency values fluctuations are caused by small amplitudes of buffeting of each sensor, the structure of which is seen as a thin flat plate, due to flow-induced forces. The study of the flow around the above plate-like structures is of great practical interest for improving the accuracy of the detection equipment.

Much theoretical and experimental effort has been expended to study the aerodynamic response of the plates and characterize the aerodynamic forces exerted on the plates. Wang and Eldredge [20] established a low-order point vortex model to investigate vortex shedding from both the trailing and leading edges of a flat plate and to predict un-steady aerodynamic forces on the plate. Fedoul et al. [21] conducted a wind tunnel experiment for the flow around a single flat plate and an array of three parallel flat plates at different angles of incidence. Their results indicated that the aerodynamic coefficient in an array of three parallel flat plates is shown to be larger than that obtained with an isolated plate only when the aspect ratio is large enough. Lin et al. [22] considered the instantaneous angle of attack for obtaining aerodynamic forces on a thin flat plate of large oscillation amplitude. They concluded that the forced torsional and vertical oscillations of large amplitude have nonlinear influence on the aerodynamic force coefficients. Bruno et al. [23] proposed an efficient method to deal with the characterization of the aerodynamic and aeroelastic behavior of a flat plate under uncertain flow conditions, and several examples of different representations of the stochastic outputs are given. Legresley et al. [24] researched the probabilistic response of a nonlinear panel in supersonic flow. They found that the panel’s response is much more sensitive to the location of the modulus fields’ extrema values than their correlation length. Poirel et al. [25] performed numerical studies for obtaining the flutter mechanism of a flexibly mounted rigid flat plate. Their results showed that the longitudinal component of turbulence lowers the flutter speed. It is believed that the decrease in flutter speed is mainly due to the small frequencies of the turbulence excitation.

For the e-nose we employed, the sensor array containing the eight sensors is fixed in a sensor box. The geometric construction of the sensor box is quite simple, but the physics describing the flow inside the box are very complex due to the three dimensional nature of flow and the interactions between the gas flow, the sensors and the sensor box’s cavity. The exact nature of gas flow inside the sensor box and the flow-induced forces on the sensors are not yet completely understood. In this paper, the realistic gas flow in the sensor box was studied utilizing CFD tools, enabling a full 3D analysis of the aerodynamic effect of the sensors on the impact of volatile gas of Chinese liquors. The aerodynamic characteristics of the eight sensors are presented and the influence on the resonance frequency values fluctuations by the flow-induced forces is evaluated. In this paper, we also employed the following nomenclature, as listed in Table 1.

## 2. Materials and Methods 

### 2.1. Novel Polymer Piezoelectric Sensor Electronic Nose (E-Nose)

**Figure 1:**
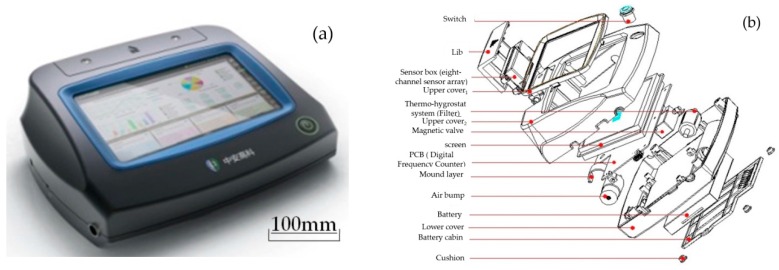
(**a**) Image of the e-nose; (**b**) Assembly diagram of the e-nose.

**Figure 2:**
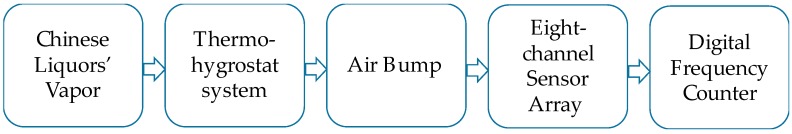
The working flow chart of the e-nose.

The novel polymer piezoelectric sensor electronic nose (E-nose) in this study, that aims to acquire the testing frequency signals of Chinese liquors [18] is shown in Figure 1a. The e-nose contains a thermo-hygrostat system, an eight-channel polymer piezoelectric gas sensor array, a digital frequency counter and an air bump (model: 00H220H024, Nidec, Shenzhen, China) etc., as shown in Figure 1b. The working flow chart of the e-nose can be seen in Figure 2. The dryness index and temperature of Chinese liquors’ volatile gas are kept at a constant through the thermo-hygrostat system, and the flow velocity of the volatile gas is kept at a constant by the air bump.

**Figure 3:**
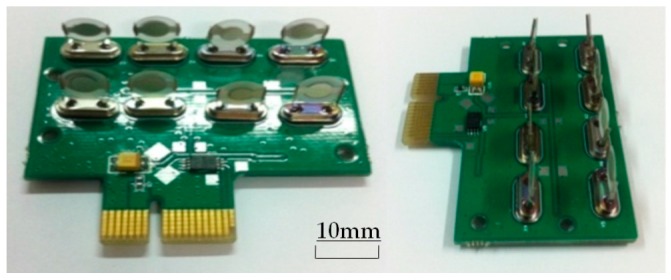
Image of the novel polymer quartz piezoelectric crystal sensor array.

The eight-channel sensor array employed in the e-nose device, as shown in Figure 3, presents feature response patterns specific to individual volatile gas due to the varying adsorptions of the Chinese liquor volatile gases on the individual array materials. A list of the eight components used in the eight-channel sensor array is given in Table 2. The resonance frequency values of all eight sensors in the array are captured by the digital frequency counter during the course of testing. Here, the resonance frequency of the sensors in the sensor array will be disturbed by the addition or removal of a small amount of mass on the polymer coating due to the adsorption and desorption of the testing materials [26]. 

### 2.2. Polymer Piezoelectric Sensors

**Figure 4:**
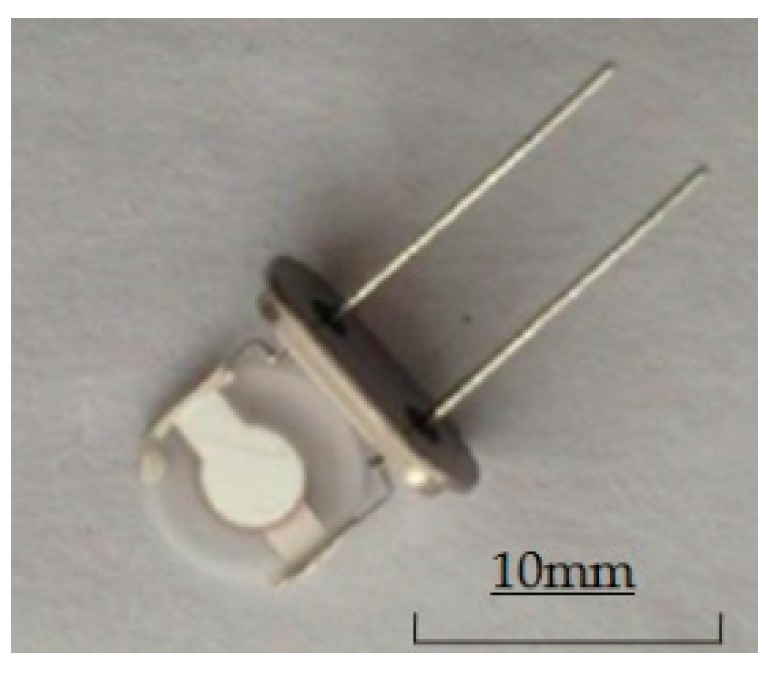
Photo of the sensor.

**Figure 5:**
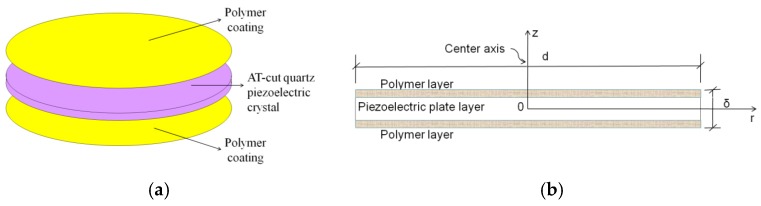
Image of the sensor’s construction. (**a**) Assembly diagram of the sensor’s construction ; (**b**) Cross-section drawn of the sensor’s construction.

A photo of the polymer piezoelectric sensor is shown in Figure 4. The sensor’s construction, which is a sandwich composite structure consisting of polymer coating/AT-cut quartz piezoelectric crystal plate/polymer coating, is displayed in Figure 5. The sensors are prepared using the Electron Beam Vacuum Dispersion (EBVD) technology [27]. The polymer coatings are sensitive components, which have absorption and desorption ability towards characteristic materials in the detecting gas [19]. The AT-cut quartz piezoelectric crystal plate is the linear electromechanical converter when the sensor vibrates according to the QCM principle [16]. The structure can achieve the goal of capturing resonance frequency signal values for gas identification. The diameter of the sensor is d = 8 mm and the sensor's thickness is δ = 0.170 mm. As transducers, they incorporate two polymer detection layers and transform an interaction, compatible reaction between the polymer layer with the volatile gas of Chinese liquors, into a testing frequency signal based on the Quartz Crystal Microbalance (QCM) principle [28].

**Figure 6:**
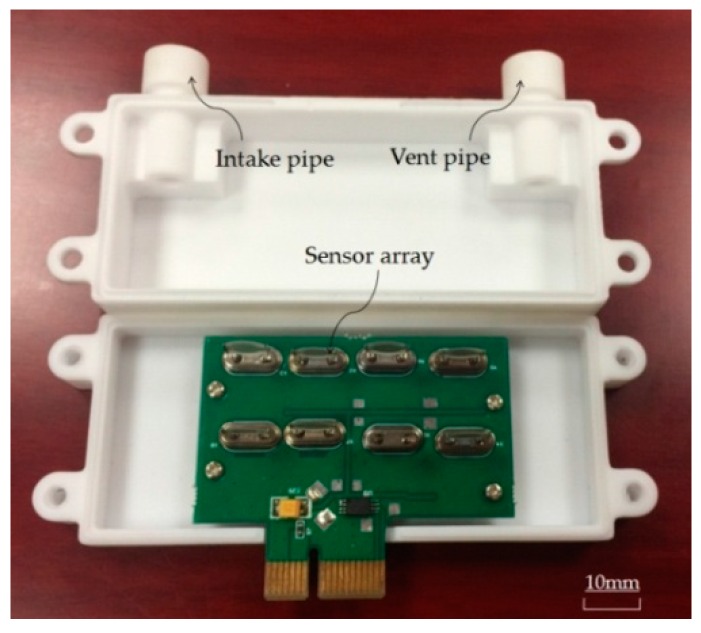
Photo of the sensor box.

The complete sensor box includes the intake pipe, the vent pipe and the sensor array, as shown in Figure 6. Volatile gases from Chinese liquors are pumped into the sensor box through an intake pipe and leave the sensor box through a vent pipe. The intake pipe and vent pipe are not completely displayed due to the dimension limits of the figure. Eight kinds of polymer quartz piezoelectric crystal sensors were employed in an eight-channel sensor array [16]. 

### 2.3. Experiments and Results

In this experiment, for Chinese liquors identification, we selected *Maotai* (Table 3), perhaps the most famous Chinese liquor, as the detection object. Experiments were performed in a stable environment at room temperature (25 °C), designed to provide a fast, non-perturbing exchange between the liquor’s volatile gas and the sensor array. The sensor box and the volatile gas were temperature stabilized to 25 ± 0.1 °C for the gas adsorption studies. Equal volumes of the volatile gas were pumped into the sensor box at a rate of 30 mL/s.

**Figure 7:**
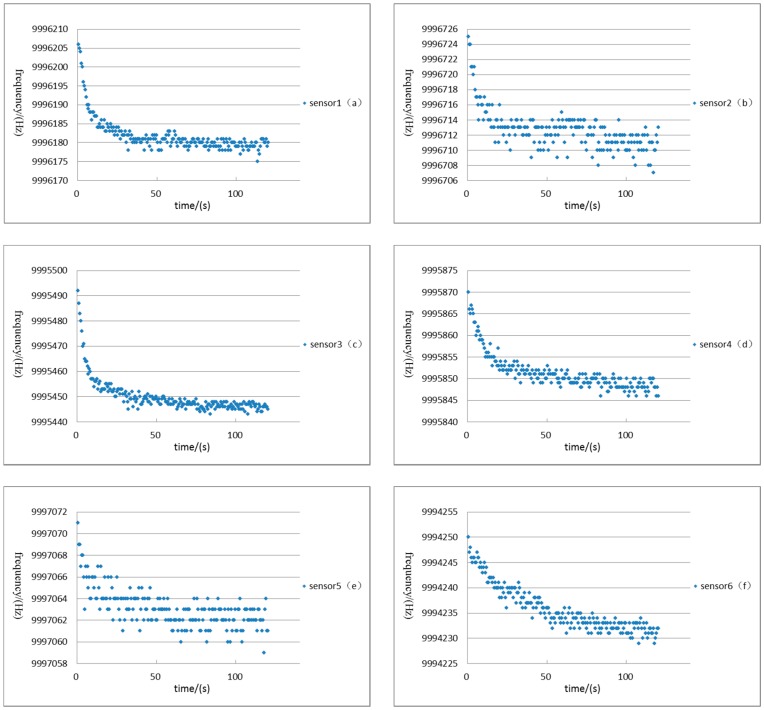

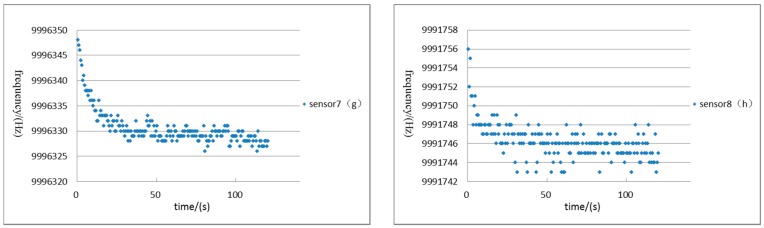
The resonant frequency values with the time extension of sensor 1–8.

The e-nose was firstly calibrated to keep the resonance frequency values of eight sensors at a constant. Two testing resonance frequency values were counted per second by the digital frequency counter. The experiment was performed 120 s, and 240 resonance frequency values were obtained. Figure 7 shows the resonant frequency values from sensor-1 to sensor-8 with the extension of time. By choosing the last 120 resonant frequency values of each sensor, we obtained the resonant frequency fluctuation of sensor 1–8 at the stable state. To present the degree of the fluctuation, the standard deviation is calculated from the 120 values (Table 4).

## 3. Assumption Define and Geometric Model

This study focuses on flow-induced forces on the sensors. The natural oscillations are high resonance frequency values from 9.991743 MHz to 9.997071 MHz. In order to model the aerodynamic forces problem, several assumptions are made here in advance. First of all, the sensor's oscillatory vibration motion is neglected; this is justifiable as a first approximation since the vibration amplitude of each sensor is relatively small at high frequencies. Then, under the above simplification, the inertia forces inherent in the sensor’s oscillatory vibration motion were not taken into account. Finally, we assume that the thickness of the sensors is negligible due to the diameter-to-thickness ratio (d/δ) of each sensor being 47 (Figure 5).

**Figure 8:**
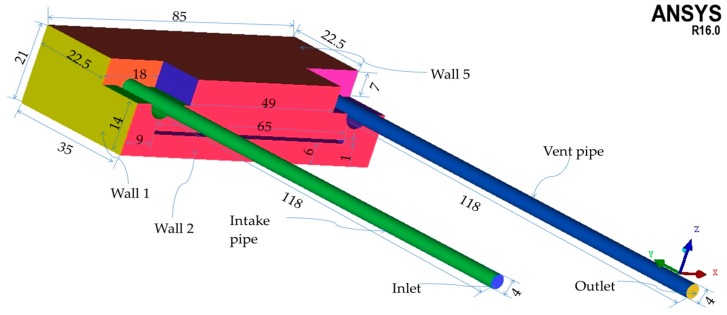
Global view of the sensor box’s cavity.

**Figure 9:**
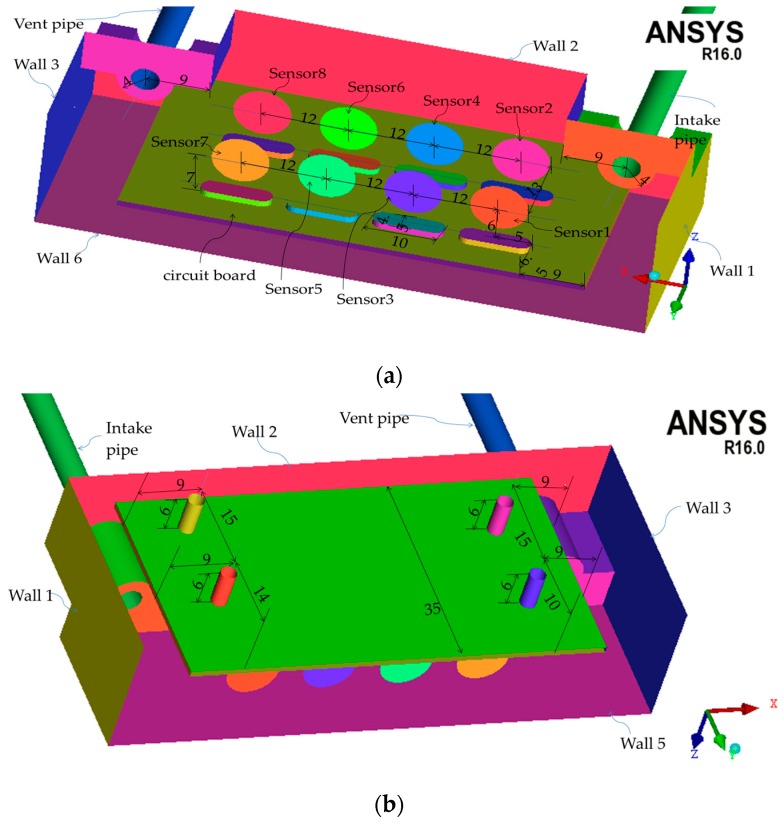
The structure in sensor box’s cavity. (**a**) Partial view 1; (**b**) Partial view 2.

To represent simply but realistically the geometry of the sensor box, a dedicated procedure was conducted for the reconstruction of the 3D sensor box model from the available database. Figure 8 shows the dimensions of sensor box's cavity from a global-view image. Figure 9 also shows the global coordinates of the sensor box model. The global coordinates have x axis normal to the “Wall 1”, y axis perpendicular to the “Wall 2”, and z axis normal to the “Wall 5”. The origin of global coordinates is fixed at the point “o”. Figure 9a,b display the dimensions of the structures in sensor box’s cavity from two partial-view images. “Wall 4” is not presented in either of Figure 8a,b in order to display the structures clearly.

The principal components of the *Maotai* are water and alcohol. The determining factor of the *Maotai* flavor are the complex trace organic compounds present in this Chinese liquor, such as ethyl palmitate [29]. In order to reduce the indispensable computation process for further modeling, the volatile gas of *Maotai*, the volatile gas of Chinese liquor, could be assumed as a mixture of gases containing 53% (mole fraction) alcohol vapor, 46.5% (mole fraction) water vapor and 0.5% (mole fraction) ethyl palmitate (C_18_H_36_O_2_) gas.

## 4. Modeling

### 4.1. Governing Equations in Scenario

The governing equations are the conservations of mass, momentum, energy and species diffusion, are as follows:

(1) $$\frac{\partial u}{\partial x}+\frac{\partial v}{\partial y}+\frac{\partial w}{\partial z}=0$$

(2) $$\frac{\partial u}{\partial t}+u\frac{\partial u}{\partial x}+v\frac{\partial u}{\partial y}+w\frac{\partial u}{\partial z}=-\frac{1}{\rho }\frac{\partial p}{\partial x}+\frac{\mu }{\rho }\left(\frac{\partial^{2}u}{\partial x^{2}}+\frac{\partial^{2}u}{\partial y^{2}}+\frac{\partial^{2}u}{\partial z^{2}}\right)$$

(3) $$\frac{\partial v}{\partial t}+u\frac{\partial v}{\partial x}+v\frac{\partial v}{\partial y}+w\frac{\partial v}{\partial z}=-\frac{1}{\rho }\frac{\partial p}{\partial y}+\frac{\mu }{\rho }\left(\frac{\partial^{2}v}{\partial x^{2}}+\frac{\partial^{2}v}{\partial y^{2}}+\frac{\partial^{2}v}{\partial z^{2}}\right)$$

(4) $$\frac{\partial w}{\partial t}+u\frac{\partial w}{\partial x}+v\frac{\partial w}{\partial y}+w\frac{\partial w}{\partial z}=-\frac{1}{\rho }\frac{\partial p}{\partial z}+\frac{\mu }{\rho }\left(\frac{\partial^{2}w}{\partial x^{2}}+\frac{\partial^{2}w}{\partial y^{2}}+\frac{\partial^{2}w}{\partial z^{2}}\right)$$

(5) $$\frac{\partial \left(c_{p}T\right)}{\partial t}+u\frac{\partial \left(c_{p}T\right)}{\partial x}+v\frac{\partial \left(c_{p}T\right)}{\partial y}+w\frac{\partial \left(c_{p}T\right)}{\partial z}=\frac{\partial }{\partial x}\left(k\frac{\partial T}{\partial x}\right)+\frac{\partial }{\partial y}\left(k\frac{\partial T}{\partial y}\right)+\frac{\partial }{\partial z}\left(k\frac{\partial T}{\partial z}\right)$$

(6) $$\begin{array}{c} \frac{\partial Y_{i}}{\partial t}+u\frac{\partial Y_{i}}{\partial x}+v\frac{\partial Y_{i}}{\partial y}+w\frac{\partial Y_{i}}{\partial z}=\frac{\partial }{\partial x}\left(D_{i,eff}\frac{\partial Y_{i}}{\partial x}\right)+\frac{\partial }{\partial y}\left(D_{i,eff}\frac{\partial Y_{i}}{\partial y}\right)+\frac{\partial }{\partial z}\left(D_{i,eff}\frac{\partial Y_{i}}{\partial z}\right)\\ \text{where}\;i=1,\;N-1 \end{array}$$

Also, assuming that the variations of temperature across the sensor box are negligible, the mixture density is calculated based on the ideal mixture state [30] at the working temperature and pressure:

(7) ρ=pop∑iRTopYiMi

Other thermo-physical properties of the mixture such as constant-pressure specific heat, thermal conductivity, and dynamic viscosity are calculated based on the mixture composition, as stated in [30,31].

### 4.2. CFD Approach

The governing equations of the gas flow in sensor box were discretized by means of the finite volume method. The discretized algebraic equations were solved iteratively by using the unstructured CFD solver, Fluent 16.0 (ANSYS, Pittsburgh, PA, USA). The software ICEM (ANSYS, Pittsburgh, PA, USA) was used as a preprocessing tool of Fluent for mesh generation.

### 4.3. Mesh Generation

**Figure 10:**
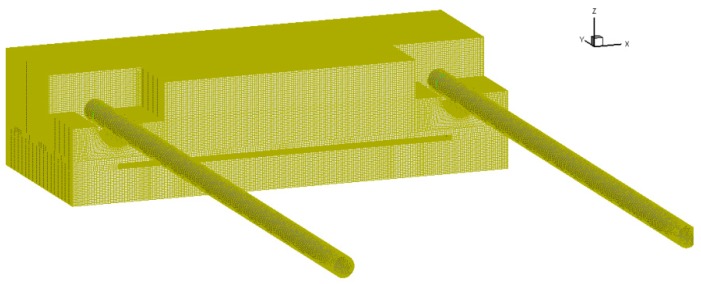
Schematic of block structured mesh used in this study.

The mesh of the computational domain generated by ICEM was shown in Figure 10. A partial-view image of the mesh could be seen in Figure 10. The sensor box’s cavity was decomposed into seven blocks so as to generate a fine structural hexahedral mesh. The generated mesh had maximum density near each sensor, which decreases smoothly towards the wall of the cavity. The mesh design of 2,529,639 nodes was chosen for evaluation of flow-induced forces on each sensor after performing steady simulation for a grid independence study.

### 4.4. Boundary Conditions

The velocity of the inlet boundary condition was implemented at the inlet of the sensor box and a presumed uniform inlet velocity, 2.4 m/s, corresponding to the real situation at the inlet boundary condition. The type of outlet boundary was set as the pressure-outlet boundary where relative pressure was set to zero in accordance with the real environment. Both the inlet stream and the outlet stream temperature are maintained at 25 °C (room temperature), and the sensor box is fixed at a constant temperature. Furthermore, the gas flow does not slip onto the walls of the sensors and the walls are impermeable. The walls are also held at a constant temperature. Identical wall boundary conditions were also applied at the walls of the sensor box’s cavity. 

### 4.5. Simulation Strategy

The steady simulation was utilized for studying the grid independence and the unsteady simulation was adopted to model the gas flow in the sensor box, respectively. However, in both the approaches, the steady state solver was initially run for 2000 iterations with de-creased Under Relaxation Factors (URF) from which residuals start exhibiting periodical oscillations. The steady simulation was then solved by further running the steady solver until the monitored facet average static pressure that passed through both the inlet and outlet became stable. The unsteady simulation was run by cutting over to the second order implicit unsteady solver with original default URF and constant time step size of 0.0004 s. The transient runs required 10–20 iterations per time step to reach the desirable residual value 1 × 10^−4^. A total number of time steps of 37,500 was used for the full unsteady simulation from 0 s to 15 s.

### 4.6. The Grid Independence Study

The computational results are greatly influenced by the grid, so the grid independence study should be able to acquire accurate simulation results. The grid independence study was performed by steady simulation. Three levels of mesh containing 2,026,140, 2,529,639 and 3,018,395 cells, for modeling gas flow in the sensor box have been tested, to be sure that the obtained results are grid-independent. The computational results of the three grid types are examined in Table 5. As seen in the table, the maximum difference between the coarsest and finest grid is less than 1%, so the 2,026,140 cells grid produces grid-independent results. To exclude any uncertainty, computations have been performed using the 2,529,639 cells grid, where the total number of cells was not critical with respect to the computation overhead.

## 5. Results and Discussion

### 5.1. Velocity Streamlines Distribution

**Figure 11:**
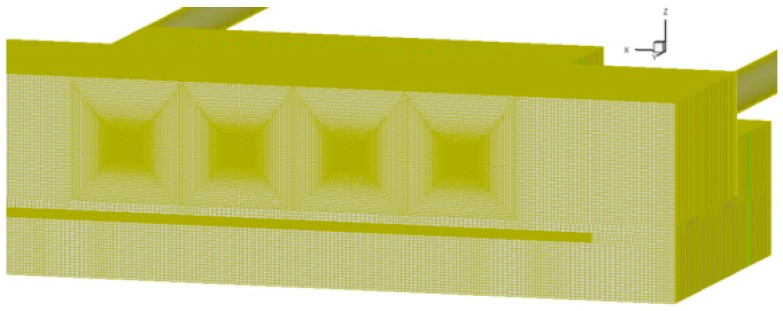
Schematic of block structured mesh from a partial-view image.

**Figure 12:**
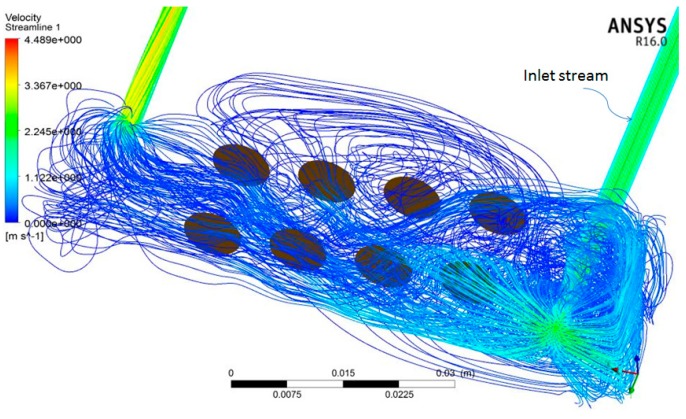
Velocity streamlines distribution of the gas flow relative to the sensors at 1 s.

Figure 11 shows the schematic of block structured mesh from a partial-view image. The velocity streamlines distribution of the gas flow in the sensor box obtained from the simulation results at 1 s is shown in Figure 12, where red lines (in web version) signify high velocity, and blue lines denote low velocity. The streamlines of the flow relative to the sensors, which result from the interaction among the gas flow, the sensors and the sensor box’s cavity, can be seen. It is seen that the inlet stream impacts with the wall, forming a strong swirling flow which would result in intense pressure fluctuations near the intake tube in the sensor box.

### 5.2. Static Pressure Distribution

**Figure 13:**
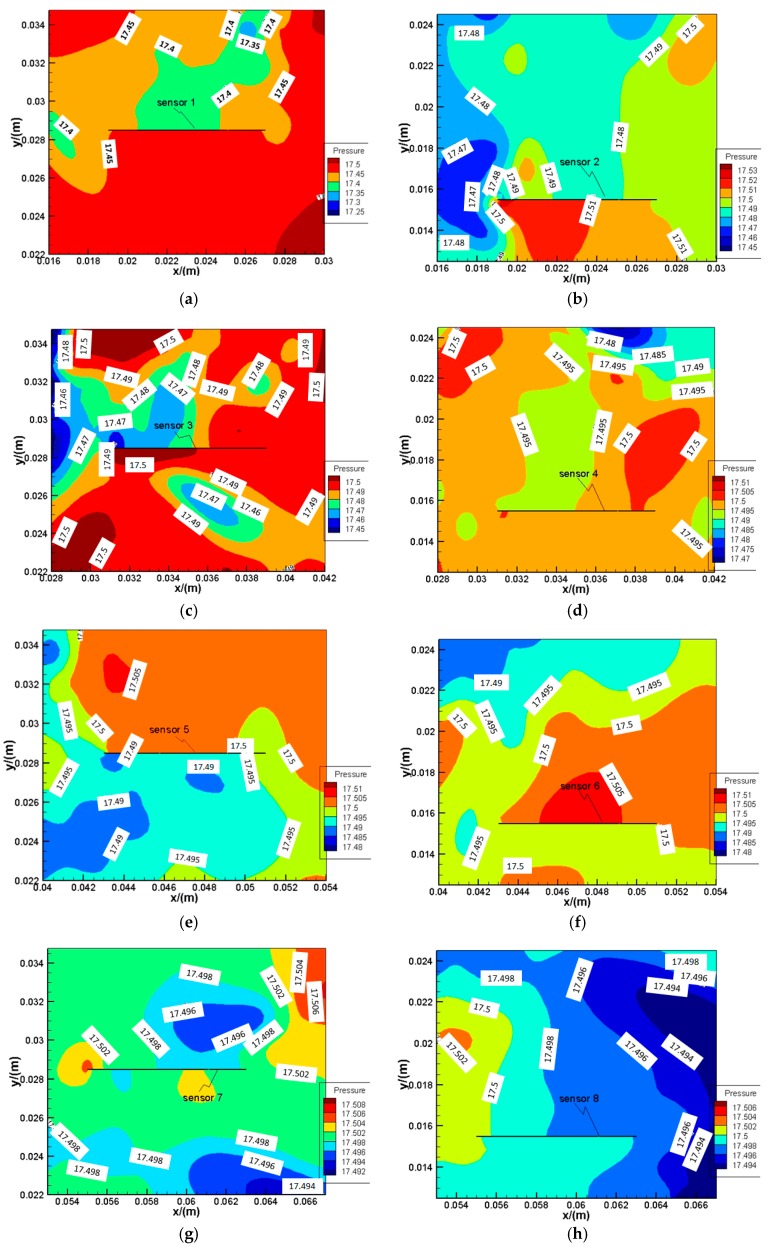
Contours of the static pressure distribution near each sensor on section z = 0 at 1 s. (**a**) Sensor 1; (**b**) Sensor 2; (**c**) Sensor 3; (**d**) Sensor 4; (**e**) Sensor 5; (**f**) Sensor 6; (**g**) Sensor 7; (**h**) Sensor 8.

To present the aerodynamic characteristics near each sensor, the static pressure distribution at axial station (with respect to global coordinates in Figure 7) located at z = 0 (including the center axis of the eight sensors, in Figure 4) from “Wall 5” is used. Eight partial-view sections are applied to plot the contour of the static pressure distribution on section z = 0 at 1 s as shown in Figure 13a–h. As seen from the figure, near one side of each sensor, a low pressure zone exists, while on another side a relative high pressure zone exists. This means there was impact effect on the sensor which causes impact loss in the sensor box. The impact effect would lead to minor deformations of the sensors, which result in the resonance frequency values' shift from their natural frequency value.

### 5.3. Characteristics of Flow-Induced Forces

The steady state simulation was utilized for studying the grid independence and the unsteady simulation was adopted to model the gas flow in the sensor box, respectively. Since the flow in the sensor box has a time-varying nature and is three dimensional and complex, the flow-induced forces on each sensor have corresponding characteristics. 

**Figure 14:**
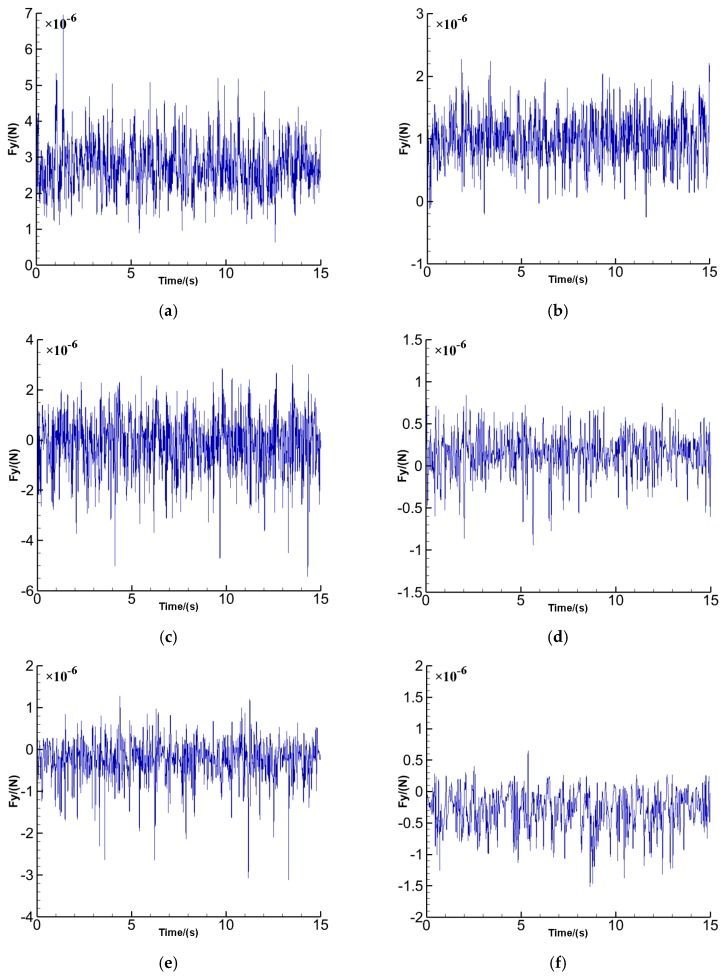

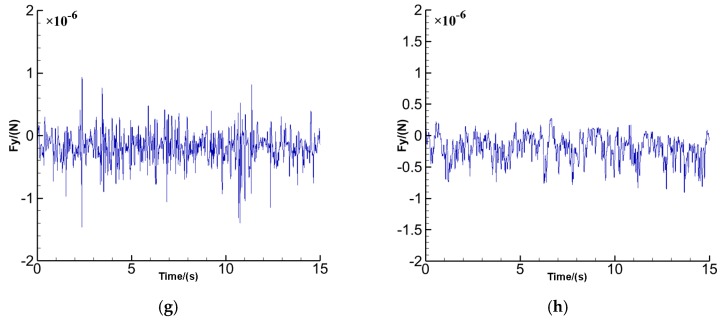
Timelines of F_y_ (**a**) Sensor 1; (**b**) Sensor 2; (**c**) Sensor 3; (**d**) Sensor 4; (**e**) Sensor 5; (**f**) Sensor 6; (**g**) Sensor 7; (**h**) Sensor 8.

**Figure 15:**
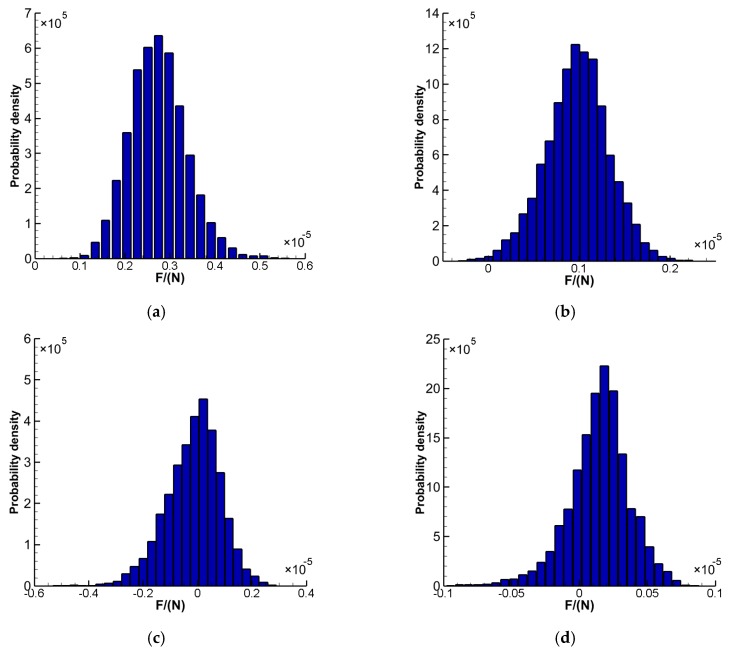

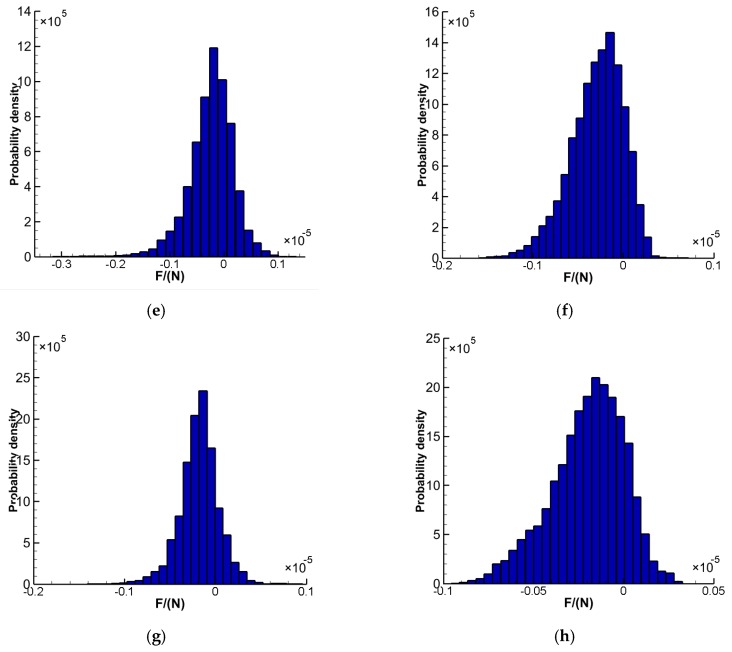
Probability density distribution of F_y_ (**a**) Sensor 1; (**b**) Sensor 2; (**c**) Sensor 3; (**d**) Sensor 4; (**e**) Sensor 5; (**f**) Sensor 6; (**g**) Sensor 7; (**h**) Sensor 8.

To display the time-varying aerodynamic forces on the sensors, 37,500 resultant force (F_y_) values on each sensor in the y-direction (with respect to the global coordinates of Figure 7) generalized during the numerical computations from 0 s to 15 s are demonstrated in Figure 14 a–h. The resultant force (F_y_) values are recorded every time step. The F_y_ on each sensor did not exhibit distinct periodical fluctuations. Probability density distributions of the fluctuating F_y_ are illustrated in Figure 15 a–h, where the probability density of F_y_ on the sensors may be described by the standardized Gaussian distribution. 

In order to evaluate the degree of the resonance frequency value fluctuations under unsteady gas flow around each sensor, the fluctuations of F_y_ which can be mathematical expressed by the standard deviation of F_y_, (see Equation (8)). The obtained standard deviation of F_y_ from this equation are listed in Table 6.

(8) $$SD_{F_{y}}=\sqrt{\frac{1}{N}\sum_{i=1}^{N}{\left(F_{y,i}-\bar{F_{y}}\right)}^{2}}$$

where N is 37,500. F_y,I_ is the F_y_ values being recorded and $\bar{F_{y}}$ is the time average values from 0 s to 15 s.

**Figure 16:**
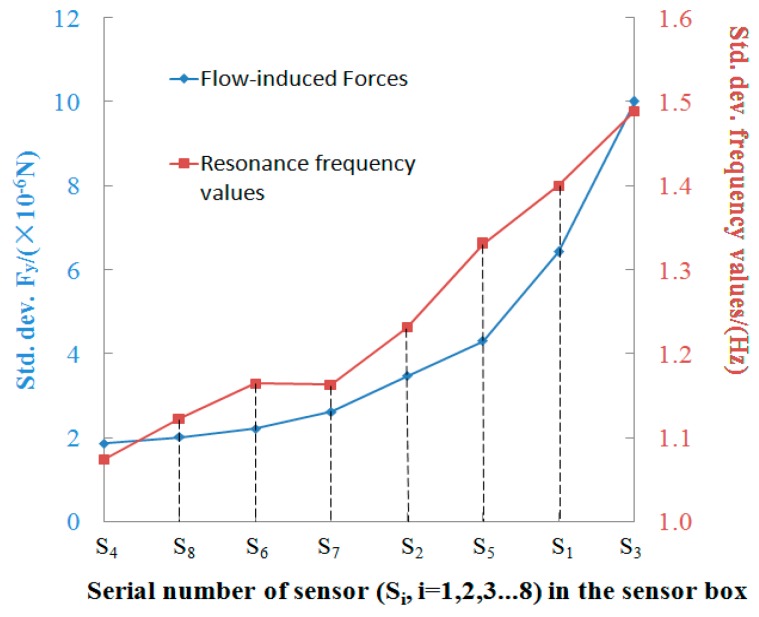
Blue line chart: Standard deviation of flow-induced force (F_y_) of each sensor. Red line chart: Standard deviation of frequency values of each sensor.

Figure 16 shows the standard deviation values of flow-induced force (Fy) of each sensor and the standard deviation values of frequency values of each sensor. The standard deviation is used to assess how variable the flow-induced force (F_y_) on each sensor and the resonance frequency value of each sensor. As we can see, the two groups of standard deviation values have the same distribution trends on the eight sensors. When the fluctuations of F_y_ are stronger, the fluctuations of the resonance frequency values are more obvious. However, when the fluctuations of F_y_ are not strong, the fluctuations of the resonance frequency values are affected slightly. In particular, both the ${\text{SD}}_{F_{y}}$ and the frequency fluctuation change of the “sensor 3” are the most notable. The frequency signals of “sensor 3” would be influenced heavily by the flow-induced force under the condition of the designed distribution of the sensors in the sensor box shown in the Figure 6 and Figure 8.

This means the effect of the flow-induced force, produced due to the distribution of sensors in the sensor box, is an important factor for obtaining the frequency values accurately, so the distribution of the sensors in the sensor box should be considered when we design a novel e-nose for identifying Chinese liquors accurately.

## 6. Conclusions

A 3D CFD simulation using the finite volume method is employed to study the flow-induced forces on each sensor in a sensor box. A dedicated procedure was developed for modeling the flow of volatile gas of *Maotai* (the most famous Chinese liquor) in a realistic scenario to give reasonably good results with fair accuracy. The simulated results illustrated that a strong swirling flow is formed near the intake tube, which is explained by the inlet stream's impact on the wall. The resultant force ($F_{y}$) values on each sensor in the y-direction were recorded at every time step during the numerical simulation. Under the influence of the gas flow, the probability density distributions of the F_y_ on each sensor are close to the standardized Gaussian distribution. We obtained the standard deviation (${\text{SD}}_{F_{y}}$) of the F_y_ from “sensor 1” to “sensor 8”. Comparing the standard deviation between the F_y_ and the resonance frequency values of each sensor, the results show that the fluctuations of F_y_ are bound up with the resonance frequency values fluctuations. When the fluctuations of F_y_ are stronger, the fluctuations of the resonance frequency values are more obvious. However, when the fluctuations of F_y_ are not strong, the fluctuations of resonance frequency values are affected slightly. The conclusion shows that, for the resonance frequency values fluctuation of the sensors at stable state, the influence of the flow-induced forces should not be neglected. To ensure that the sensor's resonance frequency values are steady and do not fluctuate significantly, in order to improve the identification accuracy of Chinese liquors using the e-nose, the sensors in the sensor box should be in the proper place, i.e., where the fluctuations of the flow-induced forces is relatively small. The results play a significant reference role in determining the optimum design of an e-nose for accurately identifying Chinese liquors.

## Figures and Tables

**Table 1:** Nomenclature.

Nomenclature	
*t*	time, s
*x*	*x*-th component of the coordinate vector, m
*y*	*y*-th component of the coordinate vector, m
*z*	*z*-th component of the coordinate vector, m
*u*	*x*-th component of the velocity, m/s
*v*	*y*-th component of the velocity, m/s
*w*	*z*-th component of the velocity, m/s
*p*	pressure, Pa
*T*	temperature, °C
*c_p_*	constant pressure specific heat, KJ/(kg·°C)
*c_T_*	heat exchange coefficient, W/(m·°C)
*R*	universal gas constant, J/(kmol·°C)
*D*	mass diffusivity, m^2^/s
*F*	force, N
*Greek Symbols*	
*ρ*	density, kg/m^3^
*μ*	dynamic viscosity, N·s/m^2^
*χ*	mole fraction
*Subscripts*	
*op*	operating condition
*eff*	effective
*mix*	mixture

**Table 2:** List of the eight polymer components employed in the 8-channel sensor array.

Number	Components	Number	Components
Ch1	PVC	Ch5	PE + AgCl
Ch2	Polyamide	Ch6	Azithromycin
Ch3	Polyethylene (PE) + AgCl	Ch7	CuCl_2_ + PE
Ch4	Polytef	Ch8	PE + CuCl_2_ + AgCl

**Table 3:** The parameters of *Maotai* (the most famous Chinese liquor).

Name	Flavor	Proof	Production Date [year]	Origin
*Maotai*	Soy sauce flavor	106	2001	Matai village, Guizhou province

**Table 4:** Standard deviations of resonance frequency values on each sensor in realistic scenario.

Sensor	Sensor-1	Sensor-2	Sensor-3	Sensor-4	Sensor-5	Sensor-6	Sensor-7	Sensor-8
SD_F_/(Hz)	1.400290853	1.231276931	**1.488870946**	1.074123202	1.330487633	1.164281037	1.162889042	1.122690596

**Table 5:** The details of the grid independence study for the sensor box’s cavity.

Total Number of Cells	Static Pressure Drop [N/m^2^]	Total Pressure Drop [N/m^2^]
2,026,140	29.001146	27.84077
2,529,639	29.243730	28.08658
3,018,395	29.065234	27.90708
% Difference ^a^	0.22	0.24

^a^ The percentage difference between the coarsest and finest grid.

**Table 6:** Stand deviations of F_y_ on each sensor.

Sensor	Sensor-1	Sensor-2	Sensor-3	Sensor-4	Sensor-5	Sensor-6	Sensor-7	Sensor-8
Std. dev.F_*y*_/(10^−7^ N)	6.42289	3.46373	10.0068	2.26483	4.30380	2.92119	2.21538	2.01107
